# Supplementary material for: STRIPAK, a Key Regulator of Fungal Development, Operates as a Multifunctional Signaling Hub
Source: J Fungi (Basel). 2021 Jun 1;7(6):443. doi: 10.3390/jof7060443 (PMC8226480; doi:10.3390/jof7060443)
Supplement: Supplementary file 1 [file jof-07-00443-s001.zip › jof-1217498-supplementary.pdf]

Supplementary material – to Perspective submitted to Journal of Fungi.

STRIPAK, a key regulator of fungal development, operates as a multifunctional signaling hub

Ulrich Kück[1#] and Valentina Stein[1]

[1]Allgemeine und Molekulare Botanik, Faculty for Biology and Biotechnology, Ruhr-University, 44780 Bochum, Germany

[#]Corresponding author: ulrich.kueck@rub.de

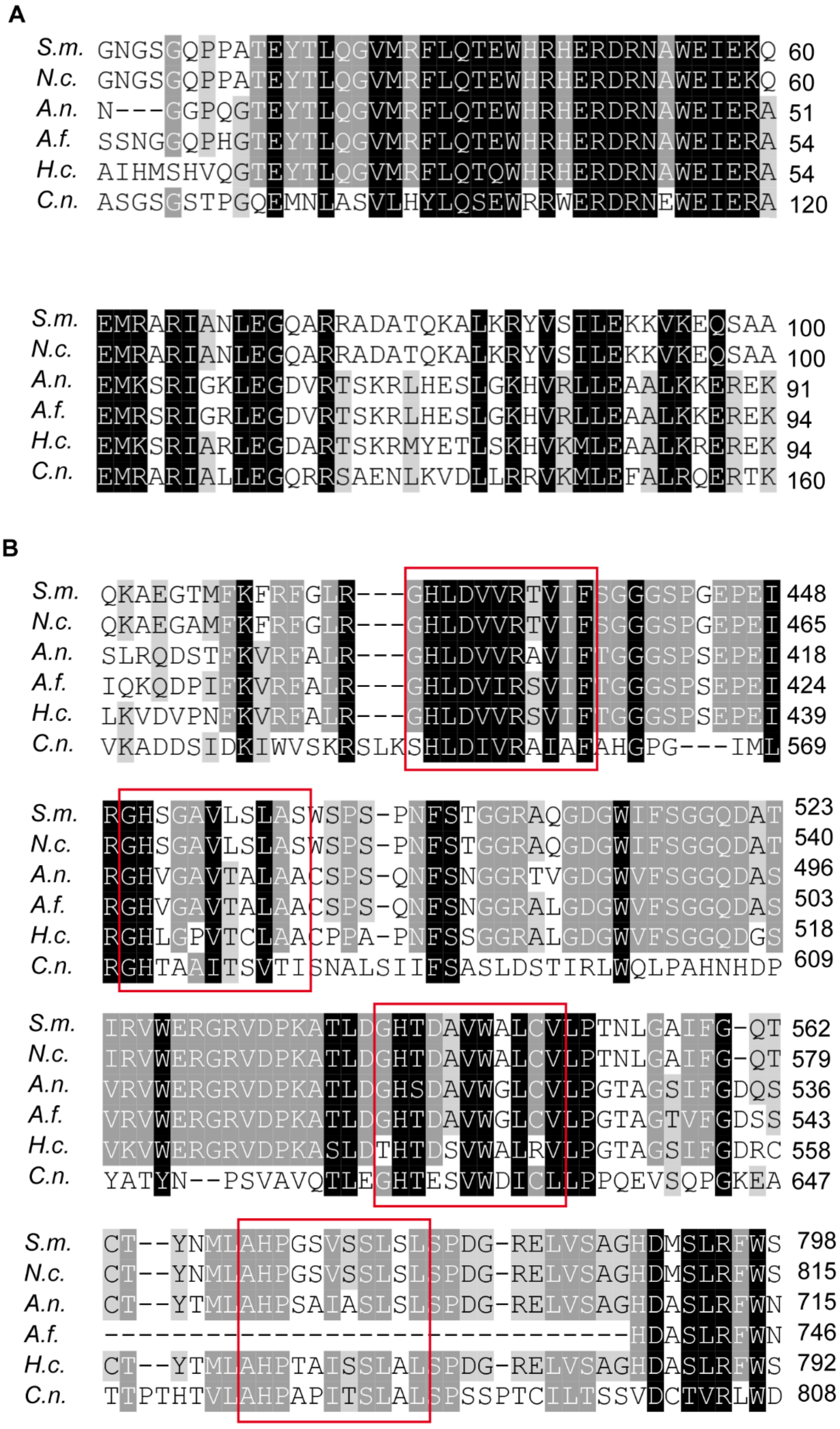

Figure S1: Alignment of striatin homologues in selected fungi. (A) Coiled-coil domain. (B) Examples of WD40 domains are framed in red. Consensus sequence of WD40 domain is (G/S/A/V)HxxxΦxx(Φ/S). X, any amino acid; Φ, any hydrophobic residue (1-3). Numbers indicate number of the last amino acid of each row. *S.m., Sordaria macrospora; N.c., Neurospora crassa; A.n., Aspergillus nidulans; A.f., Aspergillus fumigatus; H.c., Histoplasma capsulatum; C.n., Cryptococcus neoformans.*

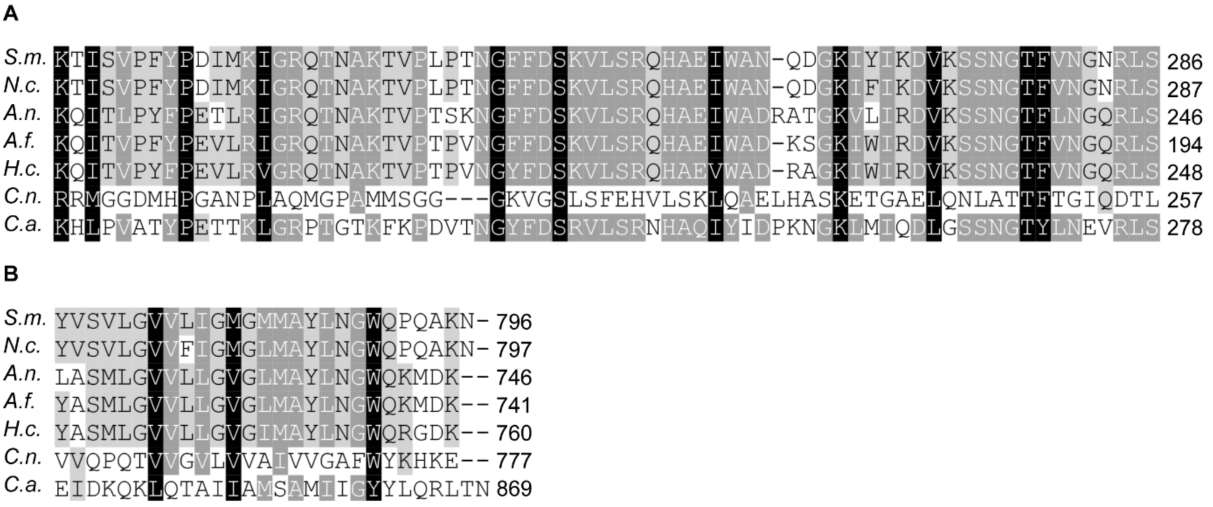

Figure S2: Alignment of SLMAP homologues in selected fungi. (A) fork-head associated (FHA) domain. (B) Transmembrane domain. Numbers indicate number of the last amino acid of each row. *S.m., Sordaria macrospora; N.c., Neurospora crassa; A.n., Aspergillus nidulans; A.f., Aspergillus fumigatus; H.c., Histoplasma capsulatum; C.n., Cryptococcus neoformans.*

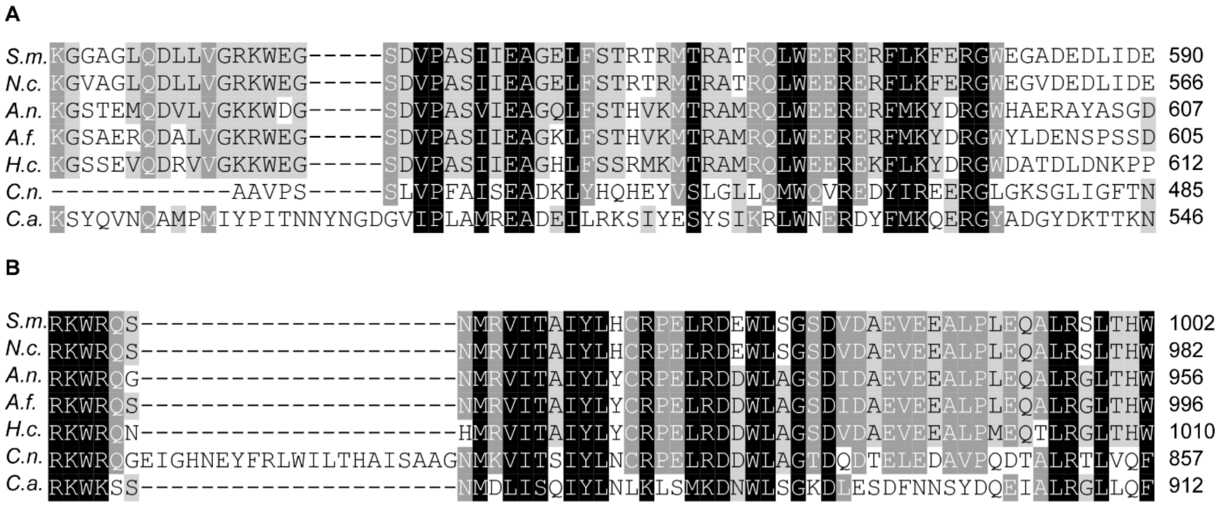

Figure S3: Alignment of STRIP1/2 homologues in selected fungi. (A) N- and (B) C-terminal part of the DUF3402 domain, which is characteristically found in STRIP1/2 homologues. Numbers indicate number of the last amino acid of each row. *S.m., Sordaria macrospora; N.c., Neurospora crassa; A.n., Aspergillus nidulans; A.f., Aspergillus fumigatus; H.c., Histoplasma capsulatum; C.n., Cryptococcus neoformans.*

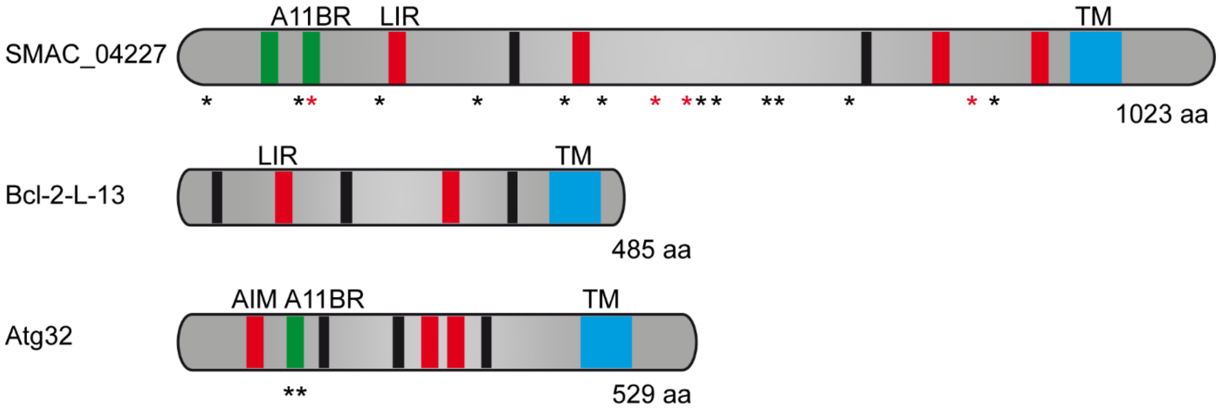

Figure S4: Domain organization of SMAC_04227 in *S. macrospora*, Bcl-2-L-13 in *H. sapiens* and Atg32p in *S. cerevisiae*. Protein SMAC_04227 was found by functional homology compared to Atg32p. Significant domains are indicted by bars: A11BR, Atg11-binding region (green), AIM, Atg8-interacting motif/ LIR, LC3-interacting region (red); amino acid acidic cluster (black); TM, transmembrane domain (blue). Phosphorylation sites in the *S. macrospora* protein are indicated by asterisks (S6, S192, S196, S234, S316, S371, S398, S415, S497, S503, S517, S567, S581, S612, S711, S732). Red stars mark differentially regulated in STRIPAK deletion mutants (S196, S415, S497, S711) (4, 5). Phosphorylation sites in *S. cerevisiae* are: S114, S119. Atg32 was adopted from and modified after Krikin et al. (2019).

Table S1: Identification of 27 putative Atg32p homologoues using eight different criteria. All predicted proteins are characterized by a C-terminal trans membrane domaine, a Casein kinase 2 phosphorylation site and a Docking site for PP2A (according to the Eukaryotic Linear Motif programme).

**SMAC***	**(F/Y/W)XX(L/I/V) Motif-LIR[1,3]**	**acidic acid clusters[1] (at least 3 acidic acids[2])**	**Exclusivley found in euascomycetes**	**A11BR[3]**	**WolfPsort prediction**
SMAC_00140	WKTL	EDD, EDE	no	SSAS	cyto 7, mito 5, nucl 4, extr 4, plas 3, pero 2
SMAC_00746	WSEI, WGEI	EED, DDD	no	SILS	plas 10, nucl 7, cyto 5, mito 4
SMAC_00819	SFVKL, STFHHV TFHHV, SSFTRL SFTRL, DFYVL DYNNL, SFTWIKV, TWIKV SSPWTDV, SPWTDV, EPSFFSV SFFSVV	EEE, EDD	no	STSS	plas 21, nucl 3, mito 2, cyto_nucl 2
SMAC_00889	WYKI, WYAI, WNSI	EEDD	no	SNSS	cyto 9.5, cyto_mito 7.5, nucl 7, mito 4.5, golg 3
SMAC_01362	WHSI, WWLL	EEE, EEEEE, DDD, DDD	no	DPSS	extr 10, cyto 6, mito 4, cyto_pero 4, E.R. 3
SMAC_02309	WYDI	EEE	no	SSSS	mito 10, nucl 9, cyto_nucl 9, cyto 7

SMAC_02807	DNFLVL	DDDD	yes	DGSS	cyto 7, nucl 6, cyto_mito 6, mito 5, plas 5
SMAC_02823	SWVEI	EED, DDD	yes	SSSS	nucl 14, cyto 6, mito 3, plas 3
SMAC_02891[4]	WEWL, WYKI, SFMNI DYISL TRSFLEV SFLEV DQSYYDL SYYDL	EEE, EEE	yes	SSLS	cyto 14.5, cyto_nucl 8, mito 6, pero 2, plas 1, extr 1, E.R. 1, vacu 1
SMAC_03631	STHFTTI, THFTTI SSVYTSL, SVYTSL	DDDDD, EDEDD	yes	DMSS	extr 20, mito 3, cyto 2
SMAC_04227[5]	SPSWEQI, SDAFSKL, SRFTSV, TYVHL	DDEEE, DEE	yes	SSSS, SILS	nucl 11.5, cyto_nucl 9, mito 6, cyto 5.5, plas 1, pero 1, golg 1, vacu 1
SMAC_04982	ETFTNI TFTNI SVFDML SGYSFI	DED	no	SESS, SCLS	plas 14, mito 5, nucl 2.5, cyto_nucl 2.5, pero 2, golg 2
SMAC_05201	WLGI, WESI	EEDD, DDEE, EDD, DDD	no	DHLS	nucl 16, cyto_nucl 12.5, cyto 7, mito 1, pero 1, cysk 1, vacu 1
SMAC_05581	WENL	EEEE , DDD	no	SILS	mito 25
SMAC_05645	DTDFTDI TDFTDI DFTDI ELYLKL	DEDEEEEE, EEE, EDD	no	SSSS	plas 9, mito 7, E.R. 7, cyto 1.5, cyto_nucl 1.5

SMAC_05985	WGSL, WENL	EED	no	STLS	mito 12, plas 5, cyto 3, nucl 2, pero 2, E.R. 2
SMAC_06013	WASL, WTIL	EED, DDDDD, DDE	no	SGSS	cyto 6.5, mito 6, cyto_nucl 6, plas 5, nucl 4.5, pero 3
SMAC_06448	WDEL	EEE	no	SRSS	cyto 14, cyto_nucl 10, nucl 4, mito 4, plas 1, extr 1, pero 1, E.R. 1, cysk 1
SMAC_06461	WALL, WLKL	EDE	no	SDSS	plas 16, nucl 4, mito 4, mito_nucl 4
SMAC_06474	WRAL	EEE, DDE	no	SSLS	mito 12, cyto 7.5, cyto_nucl 6.5, nucl 4.5
SMAC_06664	WKPI	DEE, EDD	no	SESS	extr 23, mito 2
SMAC_06939	WARI	DDE	no	SVSS	extr 21, mito 2, E.R. 2
SMAC_07007	WTDI, WKGL	EDEE, DDE, EDD	no	SKLS	plas 21, mito 4
SMAC_07048	ERGFYRV	EEE	no	AILS	plas 21, mito 4
SMAC_07073	WWVL	EEED EEE, EEE, EEE, EEE, EEE, DEE, DDE, EDD, EDD	no	SVSS	mito 8, cyto 6, cyto_nucl 6, extr 5, nucl 4, E.R. 2
SMAC_07199	WASL WAWL	EDD	no	SYSS	nucl 11, mito 5, cyto 4, plas 2, pero 2, golg 1, cysk 1, vacu 1

SMAC_08674	ECDFCSL, DDYQIL, EYVALS, DRFFTL, SKYHTV, SPEFLAV, TREWIRL	DED	yes	SISS	cyto 8, nucl 7, mito 7, mito_nucl 7

[*] no homologue in *S. cerevisiae*

[1] Murakawa et al. 2015

[2] This study

[3] Kirkin et al. 2019

[4]Protein was found in the phosphoproteome, and is regulated by STRIPAK (Märker et al. 2020, Stein et al. 2020)

[5] Protein was found in the phosphoproteome, but is not regulated by STRIPAK (Märker et al. 2020, Stein et al. 2020)

References

1. Pöggeler S, Kück U. 2004. A WD40 repeat protein regulates fungal cell differentiation and can be replaced functionally by the mammalian homologue striatin. Eukaryot Cell 3:232-40.
2. van der Voorn L, Ploegh HL. 1992. The WD-40 repeat. FEBS Lett 307:131-4.
3. Neer EJ, Schmidt CJ, Nambudripad R, Smith TF. 1994. The ancient regulatory-protein family of WD-repeat proteins. Nature 371:297-300.
4. Märker R, Blank-Landeshammer B, Beier-Rosberger A, Sickmann A, Kück U. 2020. Phosphoproteomic analysis of STRIPAK mutants identifies a conserved serine phosphorylation site in PAK kinase CLA4 to be important in fungal sexual development and polarized growth. Mol Microbiol 113:1053-1069.
5. Stein V, Blank-Landeshammer B, Müntjes K, Märker R, Teichert I, Feldbrügge M, Sickmann A, Kück U. 2020a. The STRIPAK signaling complex regulates dephosphorylation of GUL1, an RNA-binding protein that shuttles on endosomes. PLoS Genet 16:e1008819.
